# The ecological dynamics of hantavirus diseases: From environmental variability to disease prevention largely based on data from China

**DOI:** 10.1371/journal.pntd.0006901

**Published:** 2019-02-21

**Authors:** Huaiyu Tian, Nils Chr. Stenseth

**Affiliations:** 1 State Key Laboratory of Remote Sensing Science, College of Global Change and Earth System Science, Beijing Normal University, Beijing, China; 2 Centre for Ecological and Evolutionary Synthesis (CEES), Department of Biosciences, University of Oslo, Blindern, Oslo, Norway; 3 Department of Earth System Science, Tsinghua University, Beijing, China; University of Texas Medical Branch, UNITED STATES

## Abstract

Hantaviruses can cause hantavirus pulmonary syndrome (HPS) in the Americas and hemorrhagic fever with renal syndrome (HFRS) in Eurasia. In recent decades, repeated outbreaks of hantavirus disease have led to public concern and have created a global public health burden. Hantavirus spillover from natural hosts into human populations could be considered an ecological process, in which environmental forces, behavioral determinants of exposure, and dynamics at the human–animal interface affect human susceptibility and the epidemiology of the disease. In this review, we summarize the progress made in understanding hantavirus epidemiology and rodent reservoir population biology. We mainly focus on three species of rodent hosts with longitudinal studies of sufficient scale: the striped field mouse (*Apodemus agrarius*, the main reservoir host for Hantaan virus [HTNV], which causes HFRS) in Asia, the deer mouse (*Peromyscus maniculatus*, the main reservoir host for Sin Nombre virus [SNV], which causes HPS) in North America, and the bank vole (*Myodes glareolus*, the main reservoir host for Puumala virus [PUUV], which causes HFRS) in Europe. Moreover, we discuss the influence of ecological factors on human hantavirus disease outbreaks and provide an overview of research perspectives.

## Introduction

Hantaviruses are enveloped RNA viruses belonging to the family Hantaviridae, genus *Orthohantavirus* [1]. They can cause serious diseases in humans, with some outbreaks resulting in case fatality rates of 12% (for hemorrhagic fever with renal syndrome [HFRS] in Europe and Asia) and up to 40% (for hantavirus pulmonary syndrome [HPS] in the Americas), depending on the hantavirus type and the resulting clinical syndrome [2,3]. Hantavirus disease came to global attention when two major outbreaks were reported during the last century. The first, an HFRS outbreak, occurred during the Korean War (1950 to 1953), when more than 3,000 United Nations troops fell ill [4]. The second was an outbreak of HPS that occurred in the Four Corners region of the southwestern United States in 1993 [5]. Hantaviruses remain a global threat to public health; they have been estimated to affect approximately 200,000 humans annually worldwide in recent years [6]. Moreover, the number of countries reporting human cases of hantavirus infection is still on the rise [7].

Human infections with hantaviruses result from contact with infected rodents or exposure to virus-contaminated aerosols; Andes virus (ANDV) is the only hantavirus in which person-to-person transmission has been documented so far [8–11]. Outbreaks of hantavirus disease are therefore considered to be associated with the primary rodent host and pathogen dynamics [12,13]. However, the mechanism of zoonotic pathogen dynamics is complex, and the relationships between or among environmental change, host–pathogen dynamics, and human spillover is far from clear [14,15]. For example, variations in incidence rates are not simply, as expected, a result of changes in rodent demography or virus prevalence [16–18]. Furthermore, although numerous research efforts have been undertaken, no WHO-licensed vaccine against hantavirus infection is available [19] (except Hantavax, which is only licensed for human use in the Republic of Korea). Current efforts to curb hantavirus transmission focus on avoiding contact between humans and host rodents [20,21]. Due to the complexity of these systems, hantaviruses deserve the attention of research scientists in the contexts of both public health and wildlife conservation.

Here, we present a review of the ecology of hantavirus diseases in an attempt to improve our understanding of the mechanisms underlying disease outbreaks. We mainly focus on three species of rodent hosts, on which there have been a wealth of longitudinal studies of population and prevalence dynamics: the striped field mouse (*Apodemus agrarius*, the main reservoir host for Hantaan virus [HTNV] [22–25]) in Asia, the deer mouse (*Peromyscus maniculatus*, the main reservoir host for Sin Nombre virus [SNV] [12,18,26–28]) in North America, and the bank vole (*Myodes glareolus*, the main reservoir host for Puumala virus [PUUV] [29–32]) in Europe. A deeper understanding of the natural ecological dynamics of host–pathogen interactions would be of great value in developing future strategies for disease prevention and control.

## Methods

### Search strategy and selection criteria

We searched the MEDLINE (via PubMed) online database and Google Scholar for articles with the key words “hantavirus,” “ecology,” or “modelling” in the title, with no date limit, published before 31 July 2017, with restriction to mainly English papers. Key words used in Medical Subject Headings were “hantavirus,” “hemorrhagic fever with renal syndrome,” “hantavirus pulmonary syndrome,” and “rodent reservoir.” Inclusion criteria were predefined as research providing information on viral infections (including human incidence, prevalence of hantavirus infections in rodent hosts, and/or host–pathogen interactions), environmental change and rodent reservoir population dynamics, and information on environmental factors that may trigger hantavirus disease outbreaks. Study data extracted included study year, location, hantavirus type, main rodent reservoir, study design, and environmental factors.

## Results

### Geographic distribution

**Fig 1:**
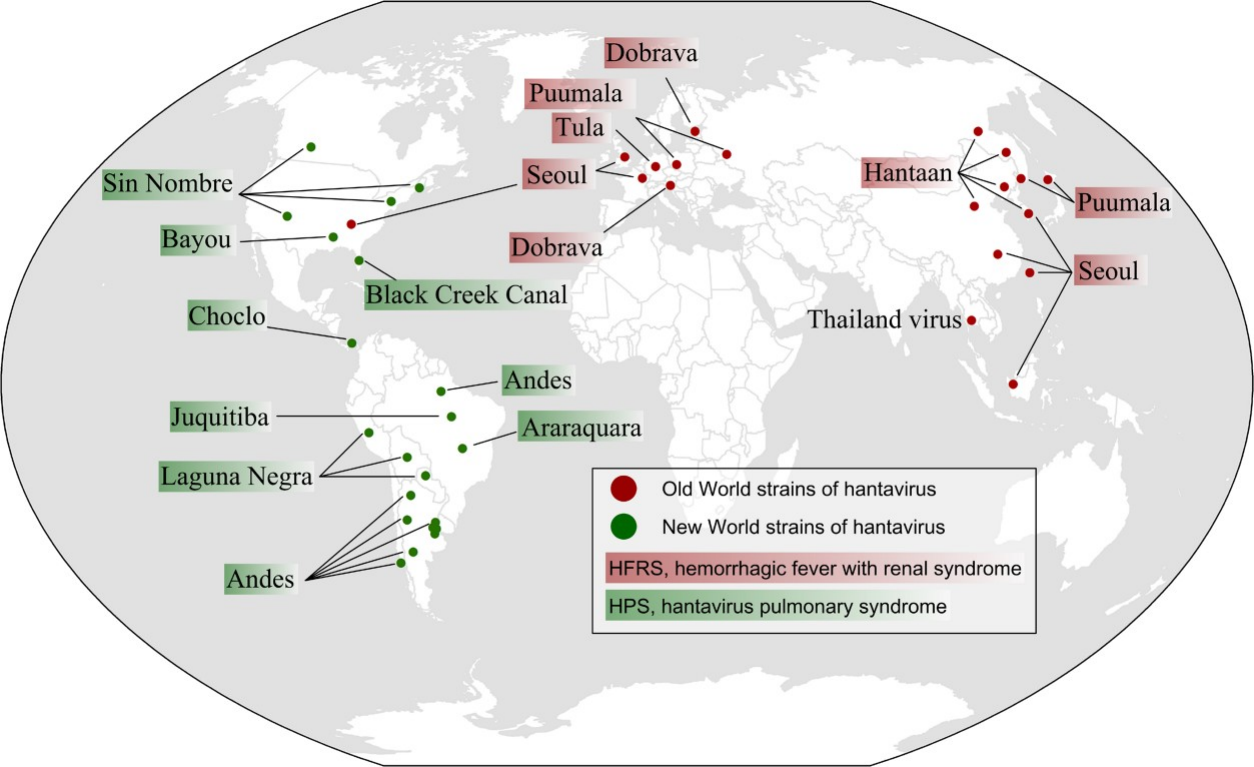
Map of Old World and New World hantavirus genotypes reported to be pathogenic for humans. Hantaviruses that have been shown to cause HFRS are shown in red, and those that cause HPS are shown in green. PUUV, which causes a milder form of HFRS (NE), is found in Europe. The described African hantavirus, Sangassou virus, was found in Guinea in 2016. In recent studies, THAIV is considered to act as an additional causative agent of HFRS. It should be noted that SEOV is harbored by *Rattus norvegicus* (brown rat) worldwide, but only those locations where reports of human infections with SEOV are shown. The map was created specifically for this manuscript and was generated by ArcGIS 9.2 (ESRI, Redlands, CA, USA) based on World Countries (http://www.arcgis.com/home/item.html?id=d974d9c6bc924ae0a2ffea0a46d71e3d). HFRS, hemorrhagic fever with renal syndrome; HPS, hantavirus pulmonary syndrome; NE, nephropathia epidemica; PUUV, Puumala virus; SEOV, Seoul virus; THAIV, Thailand virus.

Hantaviruses that cause illness in humans have been identified across the globe [3,7,33] (Fig 1). The major hantavirus disease burden in the Old World is HFRS, and in the New World it is HPS. HTNV in Asia, PUUV and Dobrava virus in Europe, and Seoul virus (SEOV) worldwide are the causative agents of HFRS. SNV, ANDV, and related viruses have been identified as causative agents of HPS in the Americas [3,12]. Recent studies indicate that the medical problem caused by hantavirus infections may be underestimated in Africa, India, Southeast Asia (where Thailand virus [THAIV] has been isolated), and even Europe [25,31,34–37] and North America [38,39]. It is estimated that hantavirus diseases are heavily underdiagnosed in Europe; only 20% of PUUV infections have been diagnosed, and no human infections data exist in several countries [31].

### Environment variation and hantavirus reservoir population dynamics

**Fig 2:**
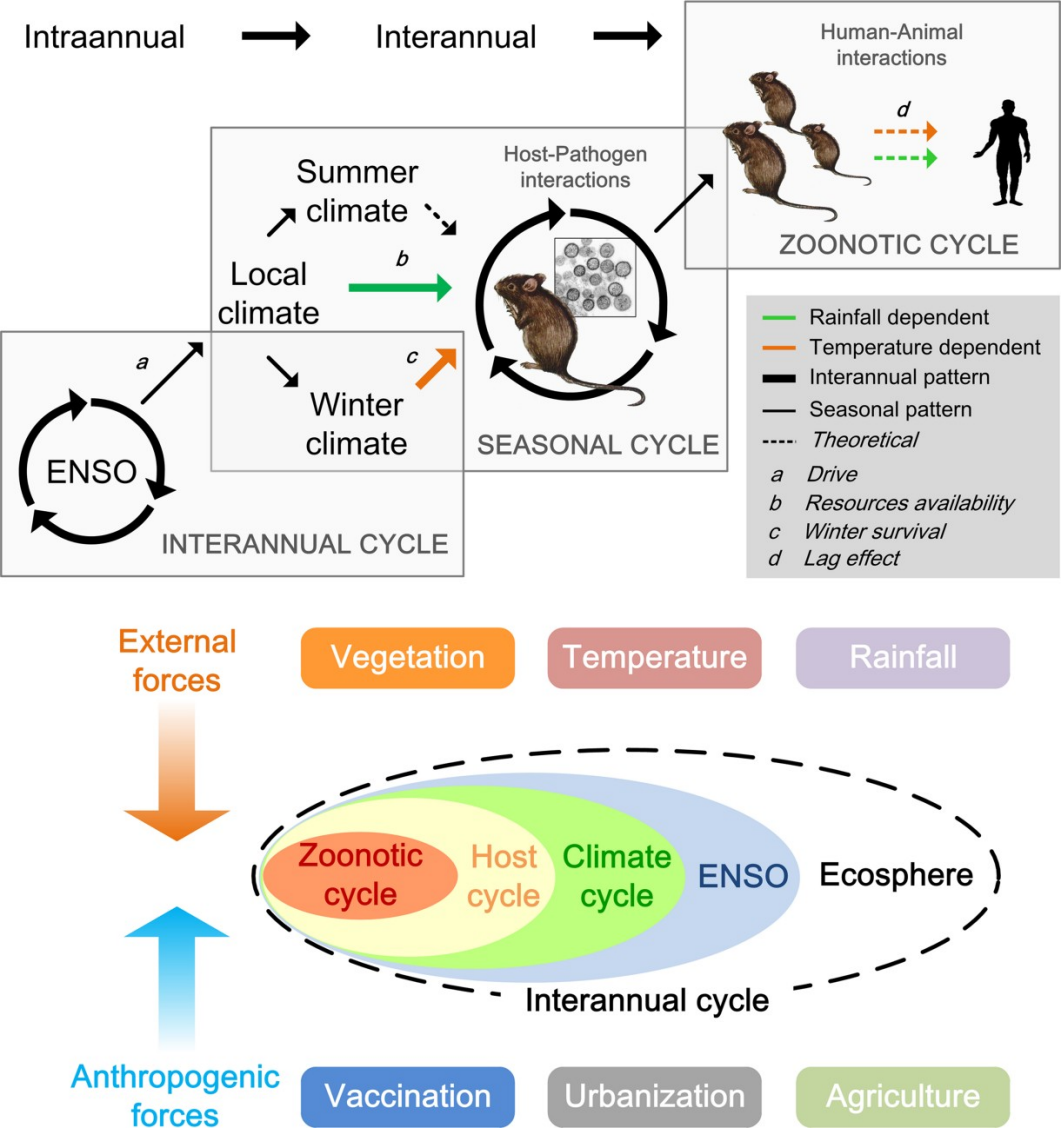
An overview of the ecological dynamics of HFRS caused by HTNV infection. Arrows represent connections affected by environment: the green line represents rainfall, and the orange line represents temperature. The solid line indicates available data, used in models linking the ENSO (Nino3.4 index) with local climate (rainfall and temperature), rodent population density (capture rate), and human HTNV infections. The rectangles delimit the seasonal, interannual, and zoonotic cycles of HTNV. Source: Adapted from [46]. ENSO, El Niño Southern Oscillation; HFRS, hemmorrhagic fever with renal syndrome; HTNV, Hantaan virus.

A bottom-up trophic cascade hypothesis has been proposed to explain the chain reactions resulting from climatic conditions, primary productivity, and host demography [27,40,41]. A bottom-up trophic cascade suggests that a change in nutrient supply could lead to similar changes in equilibrium abundances at all trophic levels [42,43]. In rodent host-hantavirus systems, climatic conditions are one of the many factors that can affect rodent population dynamics and, consequently, the prevalence of virus infection in rodent reservoirs and risk of virus exposure in humans [44] (see example shown in Fig 2). A review of the available longitudinal studies of rodent communities supports the hypothesis that hantavirus reservoir populations in both the Old World and New World are significantly influenced by climate, either directly (via winter survival) or indirectly (through food limitation).

In Central China, an environmentally induced cascading effect on the population dynamics of the striped field mouse (*A*. *agrarius*, the main reservoir host for HTNV) was found by combining 30 years of field surveillance and satellite images. The normalized difference vegetation index (NDVI) value for farmland, and precipitation two months previously, were important in determining striped field mouse survival and recruitment rates, respectively [45]. An extreme drought-induced food shortage is thought to increase mortality in striped field mouse populations, as they are a species that relies on farm crops [45]. Furthermore, winter temperature was found to exert complex effects on overwinter survival, thereby affecting the population growth rate in the following year [46]. In South China, the population density of the main reservoir host for SEOV, the brown rat (*Rattus norvegicus*), is correlated with temperature, precipitation, and the NDVI value for farmland [47]. In northeastern China, in Huludao City, an endemic area for HFRS due to SEOV, climate is considered to affect HFRS epidemics mainly through its effect on the population density of the brown rat (the most abundant rodent species present, accounting for more than 80% of the total rodent population) [48]. In Europe, food-related factors (seed production, climate affecting vegetation biomass) have been linked to rodent populations in deciduous forests [30,49]. Outbreaks of nephropathia epidemica (NE; a mild form of HFRS caused by PUUV infection) are hypothesized to have an ecological causal connection with the staple food source for the main carrier of PUUV, the bank vole, in mast years [32,50,51]. Population densities of bank voles were found to be associated with summer temperature and autumn temperature, both of which favor seed development [52,53]. A higher average winter temperature is believed to reduce winter survival of bank voles in Scandinavia because of a shorter period of protective snow cover against predators. On the contrary, increasing winter temperatures are found to provide a survival benefit to increasing populations of striped field mouse in the spring in Central China; subsequently, a large population of rodents might be accompanied by intraspecific competition due to food or space limitation, resulting in a negative-feedback effect of population density [46]. In North America, the HPS outbreak in 1993 in the Four Corners region of the southwestern US was considered to be driven by increased precipitation induced by an El Niño-Southern Oscillation (ENSO) event. The HPS outbreaks in 1997 in the same region were also preceded by an ENSO event, which brought increased precipitation, favoring deer mouse host populations [26,27]. NDVI is also used to identify locations that can be monitored for the abundance of deer mouse and presence of SNV by examining the seasonal dynamics of vegetative patterns [54]. In central Montana, US, the survival and recruitment rates of the deer mouse were found to be associated with precipitation and temperature, implying a complex relationship between climate and population dynamics [55].

### Rodent population dynamics and risk to humans

Long-term observations on the prevalence of hantavirus infection in rodent hosts are critical to understanding the dynamics of hantavirus diseases in humans. Similarly, analysis of host–pathogen interactions is important to successfully implement disease control strategies [56]. Hantaviruses are known to be directly transmitted from infected to susceptible hosts (horizontal transmission). Therefore, changes in rodent population densities are expected to increase or decrease the transmission and prevalence of hantavirus infection in rodent reservoir populations, resulting in greater or lower levels of spillover to humans [28]. The dynamics of rodent population density and hantavirus infection prevalence are linked by contact rates; the virus may become extinct below a certain host density, corresponding to the minimum number of hosts required for virus maintenance in a population [57]. A critical population density threshold of striped field mice has been observed in Central China, below which HTNV cannot invade and persist in the population [45]. A similar population density threshold has been observed in the SNV reservoir, the deer mouse, in North America [58,59]. Rodent community composition has also been found to affect the risk of human hantavirus infection among different landscapes [60].

Studies have demonstrated positive correlations between relative population density of bank voles and prevalence of PUUV infection among rodent populations [61]. Bank vole density indices were also positively correlated with risk of HFRS in humans in northern Sweden, Finland, and Norway [62–64]. However, the relationship between rodent demography and disease dynamics in China and North America is more complicated. In Central China, fluctuation in HFRS incidence is highly correlated with striped field mouse population density and the prevalence of HFRS infection in Shaanxi Province, an endemic area for HFRS due to HTNV [13]. In northeastern China, HFRS cases are associated with a virus-carrying index, an indicator to describe the combined effects of rodent population density and prevalence of virus infection, with a one-month lag, in the SEOV endemic area of HFRS [48]. However, in South China, the number of HFRS cases is not associated with rodent population density in Chenzhou and Changsha (the main species captured are *R*. *norvegicus*, *R*. *flavipectus*, and *Mus musculus*), which are mixed HTNV and SEOV endemic areas [65,66]. In North America, different relationships have been found between deer mouse population density and SNV infection prevalence in the deer mouse population—positive correlations [17,27,67], no linear correlation [59,68,69], and even negative correlations [18,70,71]. (It should also be noted that the different correlational relationship may be due to temporal issues; e.g., some studies take account of time lags when analyzing the data while others do not.) Because hantaviruses are horizontally transmitted, these inconsistent results may be due to two conflicting effects of population dynamics on prevalence [28]. During breeding seasons, rapid population growth of juveniles not yet infected may increase the proportion of uninfected rodents and potentially decrease hantavirus prevalence [72,73], whereas the resulting higher population size may eventually increase contact between individuals and prevalence. Taken together, these studies point to several issues that could be of importance to understanding the relationship between reservoir population dynamics and pathogen transmission [57]. Future demographic studies should aim to establish causal mechanisms linking behavior, demography, and virus prevalence dynamics.

### Environmental factors that trigger hantavirus disease outbreaks

Environmental factors are major triggers that affect reservoir ecology and virus ecology and thus are likely to affect hantavirus transmission from rodent reservoir to humans (or risk of virus exposure in humans). However, although outbreaks appear to be a result of these factors, it is difficult to predict the exact outcome, chiefly due to the complex and multifactorial mechanisms that drive hantavirus disease outbreaks. Studies have highlighted the importance of the interplay between extrinsic and intrinsic factors in determining hantavirus disease dynamics [45,46,74], e.g., when the size of outbreaks is small due to low population susceptibility levels as the result of vaccination or the rodent reservoir population density is below the threshold level required to maintain the virus due to environmental limitations. In this section, studies addressing environmental variability and human hantavirus infections were included (Table 1), and we mainly review three factors that play an important role in altering hantavirus disease dynamics and have been documented sufficiently—precipitation, temperature, and landscape alteration.

**Table 1:** Synthesis of the environmental factors, during the same year or before, that may trigger hantavirus disease outbreak.

Trigger factor	Detail	Disease	Area	Rodent host
**Temperature**	Summer (Y-2) and autumn temperature (Y-1)^+^	NE	Belgium [50,52], Central Europe	*M*. *glareolus*
	Winter and spring temperature (Y 0)^+^	NE	Southwestern Germany [53], Central Europe	*M*. *glareolus*
	Winter temperature−	HPS	Southern Argentina [91], South America	*Oligoryzomys longicaudatus*
	Annual mean temperature^+^	HPS	Atlantic forest, Brazil [128], South America	Family Cricetidae
	Maximum temperature (Y 0)^+^	HFRS	Heilongjiang Province, North China [129], East Asia	Unknown
	Mean temperature (Y 0)^+^	HFRS	Inner Mongolia, North China [89]; South Korea [130]; East Asia	Unknown
	Mean temperature (Y 0)^+^	HFRS	Huludao City, North China [48]; Changsha City, South China [20,66]; East Asia	*R*. *norvegicus*
	Average temperature (Y 0)−	HFRS	Shandong Province, North China [90], East Asia	Unknown
	Summer temperature (Y 0)−	HFRS	Weihe Plain, Central China [46], East Asia	*Apodemus agrarius*
**Rainfall**	Summer rainfall (Y-3)^+^	NE	Belgium [50], Central Europe	*M*. *glareolus*
	Rainfall (Y-1)^+^	HPS	the Four Corners region of New Mexico and Arizona, US [27], North America	*P*. *maniculatus*
	Winter rainfall^+^	HPS	Southern Brazil [131], South America	Unknown
	Annual rainfall^+^	HPS	Southern Argentina [91], South America	*O*. *longicaudatus*
	Monthly rainfall (Y 0)^+^	HPS	Chile [132], South America	Unknown
	Summer and autumn rainfall (Y 0)^+^; annual rainfall (Y-1)^+^	HFRS	Xi’an City, Central China [46,100], East Asia	*A*. *agrarius*
	Monthly rainfall (Y 0)^+^	HFRS	Huludao City, North China [48], East Asia	*R*. *norvegicus*
	Monthly rainfall (Y 0)^+^	HFRS	Changsha City, South China [78]; Inner Mongolia, North China [89]; South Korea [130], East Asia	Unknown
	Monthly rainfall (Y 0)−	HFRS	Jiaonan County, North China [133]; Shandong Province, North China [90]; Anhui Province, South China [85], East Asia	Unknown
**Humidity**	Absolute humidity (Y 0)^+^	HFRS	Changsha City, South China [78]	Unknown
	Relatvie humidity (Y 0)−	HFRS	Jiaonan County, North China [133]; Shandong Province, North China [90], East Asia	Unknown
	Relative humidity (Y 0)^+^	HFRS	Inner Mongolia, North China [89]; Liaoning Province, North China [134]; South Korea [130], East Asia	Unknown
	Relative humidity (Y 0)^+^	HFRS	Huludao City, North China [48], East Asia	*R*. *norvegicus*
	Temperature vegetationdryness index^+^	HFRS	Changsha City, Zhuzhou City, Xiangtan city, Hengyang City, South China [127], East Asia	*R*. *norvegicus*, *M*. *musculus*, *A*. *agrarius*, *R*. *flavipectus*
	Proportion of thin particles (<10 μm) (Y 0)^+^	NE	Northern Belgium [81], Central Europe	*M*. *glareolus*
**Air pressure**	Mean air pressure (Y 0)−	HFRS	Huludao City, North China [48], East Asia	*R*. *norvegicus*
**Air pollution**	PM_10_ (Y 0)^+^	HFRS	South Korea [130], East Asia	Unknown
**Flood**	Water-level difference of Huai River (Y 0)−	HFRS	Anhui Province, South China [86], East Asia	Unknown
**ENSO**	Southern oscillation index (Y 0)−	HFRS	Heilongjiang Province, North China [129]; Anhui Province, South China [84,135], East Asia	Unknown
	Multivariate ENSO index (Y 0)^+^	HFRS	Changsha City, South China [66,78]; Inner Mongolia, North China [89], East Asia	Unknown
	Multivariate ENSO index (Y-1)^+^	HPS	Four Corners region of New Mexico and Arizona, US [136], North America	*P*. *maniculatus*
**Land cover change**	Forestation^+^	HFRS	Liaoning Province, North China [134], East Asia	Unknown
	Developed land−; Rice paddy^+^; Orchard^+^	HFRS	Beijing, North China [119,137], East Asia	Unknown
	Area of deciduous forest^+^	NE	Northern Belgium [81,108], Central Europe	*M*. *glareolus*
	Cover of beech forest, cover of seed plant^+^	NE	Southwestern Germany [53], Central Europe; Temperate Europe [138]	*M*. *glareolus*
	Proportion of land cultivated for sugarcane^+^	HPS	São Paulo [128], South America	Family Cricetidae
	Proportion of forest cover^+^	HPS	Atlantic forest, Brazil [128], South America	Family Cricetidae
**Food availability for rodent host**	Seed production (Y-1)^+^	NE	Western and central European countries [52,53,139–141]	*M*. *glareolus*
	NDVI for trapping site (Y-1)	NE	Northern Belgium [108], Central Europe	*M*. *glareolus*
	High photosynthetic mass^+^	HPS	Southern Brazil [131], South America	Unknown
	NDVI (Y 0)^+^	NE	the Franche-Comté region, France [82], Central Europe	Unknown
	NDVI for farmland (Y 0)^+^	HFRS	Inner Mongolia [142], North China, East Asia	Unknown
	NDVI for rice paddy (Y 0)^+^	HFRS	Changsha City, South China [66], East Asia	*M*. *musculus*, *R*. *flavipectus*, *R*. *norvegicus*
	net photosynthesis (Y 0)^+^	HFRS	Weihe Plain, North China [41], East Asia	*A*. *agrarius*
**Socioeconomic factors**	Gross domestic product and the urbanization rate (Y 0)−	HFRS	Chenzhou City, South China [20], East Asia	*R*. *norvegicus*, *R*. *flavipectus*
	Human development index−	HPS	São Paulo [128], South America	Family Cricetidae
**Selenium deficiency**	Selenium content in feed−	HFRS	China [143], East Asia	Unknown

^+^Positive correlation with hantavirus infections

^−^Negative correlation with hantavirus infections

**Abbreviations:** ENSO, El Niño Southern Oscillation; HFRS, hemorrhagic fever with renal syndrome; HPS, hantavrius pulmonary syndrome; NDVI, normalized difference vegetation index; NE, nephropathia epidemica; (Y 0), during the same year; Y-n, n years before

**Fig 3:**
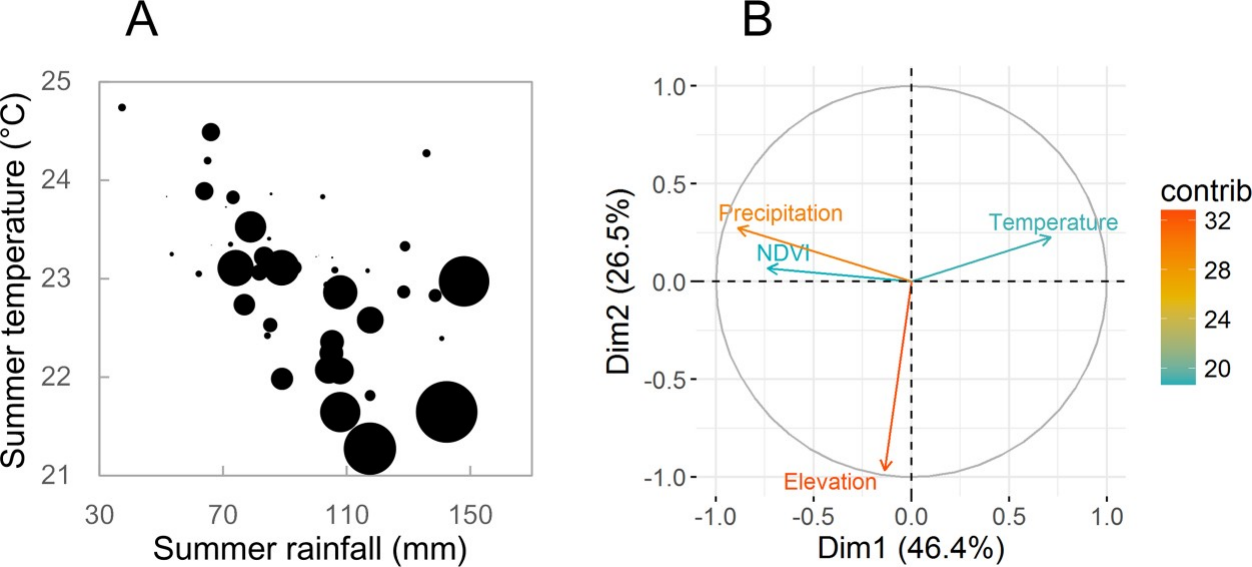
Environmental factors associated with hantavirus disease outbreaks. (A) Relationship between summer temperature, summer rainfall, and HFRS cases in Weihe Plain, North China, 1960 to 2013. Circle size is proportionate to the number of HFRS cases [46]. (B) Contribution of the environmental variables to the explained variance of hantavirus-antibody–positive in rodents using a multivariate principal component analysis in Hunan Province, South China, 2007 to 2010. Dimensions 1 and 2 are the spaces where variables are expressed. The length (angle) of the arrows represents the magnitude (direction) of the correlation coefficient between the variable and the principal components. The contributions of the variables to the hantavirus-antibody–positive in rodents are ranked with colors ranging from green to red, respectively (reproduced from Xiao 2016 with permission of the publisher [127]). Dim, dimension; HFRS, hemorrhagic fever with renal syndrome; NDVI, normalized difference vegetation index.

It is becoming increasingly more apparent that climatic variations have profound impacts on infectious disease dynamics [75,76], especially for climate-sensitive infectious diseases; e.g., human hantavirus diseases are considered climate-sensitive infectious diseases [77]. Understanding the influence of climatic drivers on hantavirus disease ecology can help in forecasting and prevention, which is even more urgently needed in this era of climate change. Recently, Tian and colleagues [46] investigated the role extrinsic factors (climatic conditions) play in determining hantavirus disease dynamics in Central China. A unique data set from Central China covering half a century showed the existence of a climate-driven transmission mechanism for HTNV from the striped field mouse to humans [21]. HFRS outbreaks were highly correlated with specific environmental conditions—low summer temperature and abundant summer precipitation. Conversely, very few disease outbreaks occurred under conditions of high summer temperatures and drought (Fig 3A) [46]. In South China, HFRS incidence was also found to be positively correlated with annual precipitation and absolute humidity during 1991 to 2010 [78]. HPS outbreaks in the Four Corners region of the US are considered to be driven by ENSO-associated precipitation events as well [26,79].

However, precipitation can also negatively affect or have no effect on the incidence of hantavirus disease, depending on magnitude and region [80]. In Europe, no clear relationship of rainfall with human hantavirus infections was shown [77], except that one study illustrated rainfall in the summer three years before hantavirus disease incidence increased [50]. Other studies also demonstrated no significant association between rainfall and hantavirus disease [52,81,82]. In Cascade and Polson, Montana, US, increases in primary productivity, deer mouse population densities, and human disease risk are less sensitive to changes in the amount of rainfall [83]. In low-lying regions of China, such as Anhui Province, HFRS incidence and rodent population density (dominated by the striped field mouse) are all negatively correlated with the total precipitation [84–86]. Heavy precipitation there may kill rodents by flooding their burrows and nests, thereby reducing host population density and the risk of human exposure to pathogens. Floods could also cause movement of rodent populations (even community-level changes [87]) to new habitats and eventually disease emergence in new sites.

Temperature could influence disease dynamics through its impact on the rodent reservoir population dynamics and pathogen survival in the external environment, subsequently influencing human–animal contact patterns. However, compared to precipitation, the effects of temperature have been less investigated, and contradictory findings make it difficult to draw firm conclusions. For example, in Central China, higher summer temperatures were followed by a lower incidence of HFRS in autumn (Fig 3A) [46]—this was presumably due to a reduced frequency of contacts between rodents and between rodents and humans, coupled with an unfavorable environment for virus survival [88]—whereas in Belgium, a higher summer temperature two years before led to higher NE incidence [50,52]. A potential explanation is that the higher temperature two years before might have stimulated bud formation that contributed to heavy masting one year before. Likewise, opposite associations between mean temperature and hantavirus infection incidence were found across China [20,66,89,90]. In South America, HPS caused by ANDV was also negatively associated with winter temperature [91]. Conversely, in North America, a positive relationship between hantavirus disease incidence and temperature was reported [80]. There is a need for more studies to investigate the mechanisms behind these relationships.

### Human–animal interface

Human–animal interface constitutes the boundary/barrier for cross species transmission of disease and the environment (including ecological and anthropological factors) within which these species exist [92,93]. In the preceding sections, we have summarized the ecological factors and processes that affect hantavirus diseases dynamics; hereafter, anthropological factors will be reviewed, including agricultural activity, human-driven land-use change, and vaccination. In HPS endemic regions of America, agriculture-associated activities were also most commonly reported as potential risk factors [94–97], and seropositive rodents were found with high frequency in agricultural landscapes [98,99]. In the HTNV-type endemic area of central China, seasonal pattern of HFRS dynamics is found to coincide with the increase in potential contact between rodents and humans in the dry season due to seasonal agricultural activities [45,100]. Besides, the breeding season of striped field mouse, the local rodent host, is closely associated with agricultural activity. Moreover, it could be concluded that agricultural activity may influence the activity and life cycles of local striped field mouse and in turn shape disease dynamics.

**Fig 4:**
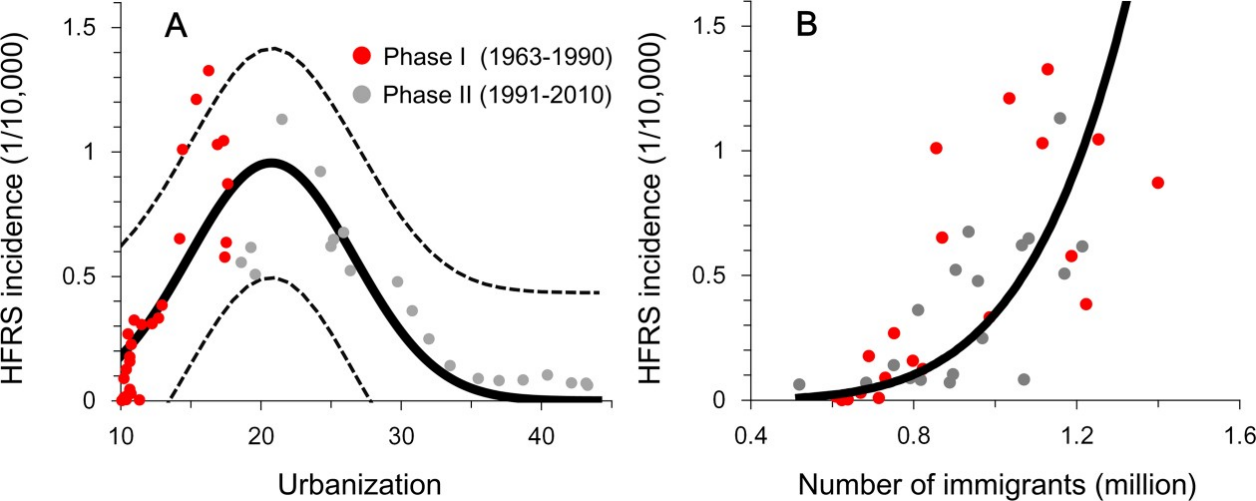
Urbanization, immigration, and hantavirus disease epidemics in an endemic area of south China. (A) Urbanization and HFRS incidence in Hunan Province. A biphasic inverted U-shaped relationship was found between hantavirus disease epidemics and urbanization. (B) The number of immigrants and HFRS incidence. This result indicates that the effect of urbanization on HFRS epidemics changed, whereas the effect of immigration remained constant. Source: Adapted from [114]. HFRS, hemorrhagic fever with renal syndrome.

Human-driven landscape change could influence rodent host behavior and the composition of reservoir communities in such a way as to impact pathogen transmission [101,102]. The major consequences of landscape alteration are habitat loss and changes in species composition due to the loss of specialist species and the increase in generalist species [74], and these consequences either increase or decrease the risk of disease transmission at the human–animal interface. For example, HFRS epidemics in China peaked in autumn and winter in the area where the dominant hantavirus is the *Apodemus*-borne HTNV, whereas the area with *Rattus*-borne SEOV saw epidemic peaks in spring [103]. These two distinct rodent species have different breeding sites with special landscape attributes [104], which changes the epidemiological characteristics of hantavirus disease. Human disturbance also affects survival probabilities and reproduction of rodent hosts [105]. Another consequence of landscape alteration is loss of biodiversity, which is considered to affect the transfer of pathogens among species and influence the risk of infection to humans. Biodiversity loss may result in increased hantavirus infection prevalence in host populations [106], affecting the dynamics of SNV in the US, Choclo hantavirus in Panama, and PUUV in Europe [68,107–111]. For some directly transmitted and vector-borne zoonotic diseases, it has been hypothesized that increased species diversity would result in a lower pathogen prevalence in competent hosts and therefore lower risk of infection to humans by a mechanism called the “dilution effect” [112], although there is still a debate about the (positive or negative) relationship between biodiversity and zoonotic disease transmission, e.g., SNV and deer mice system in America [113]. To our knowledge, few attempts have been made to evaluate this effect with regards to HTNV/striped field mouse system in China. Further testing of this theory is therefore still required. Another human-driven landscape change is induced by urbanization (i.e., urban expansion). However, the relationship between the diffusion of zoonotic pathogens and urbanization is complex because of the contrasting effects (Fig 4): cities with faster economic growth may attract more immigrants and reach their endemic turning points later (endemic turning point defined as point at which incidence changes from increasing to decreasing), whereas economic growth that contributes towards improvements in the living conditions may decrease contact between rats and humans [114].

Vaccination programs against HFRS have been in practice for more than 20 years in China, where the incidence of HFRS has decreased with occasional small fluctuations. It has been reported that the age distribution of HFRS infections has clearly changed in recent years, ever since the Expanded Program of Immunization for regions with high HFRS incidence was implemented in 2008 [115]. Simulations conducted on data from a county in Central China have shown that vaccination will alter the dynamics of HFRS outbreaks [45]. The vaccination-induced reduction in susceptible human population gradually led to the reduction of human hantavirus infections and successfully averted further epidemics. The findings in China therefore highlight the necessity for a vaccination strategy and provide important insights for other countries.

## Discussion

The dynamics of hantavirus epidemics involve multiple phases, including environmental drivers that influence infectious diseases, transmission in the animal reservoir, and spillover transmission to humans. The complexity of disease dynamics has been highlighted in recent decades by contradicting trends; the incidence of HFRS has decreased in China [22,116] and increased in Europe [31]. Although pioneering research efforts to study both New World and Old World strains of hantaviruses in rodents have been undertaken, as well as related prevention strategies, it is evident that much work remains to be done. By further studying the transmission dynamics of hantaviruses, better prediction and prevention measures can be implemented to protect human health, and critical insight can be obtained into the ecology of hantaviruses and their rodent hosts.

The geographic distributions of hantaviruses reflect the distributions of their reservoir hosts [3], and our knowledge of the host associations of hantaviruses is expanding. For example, the hantavirus isolated in Africa, Sangassou virus, was recently found in the African wood mouse (*Hylomyscus simus*) [117]. Although, in general, each hantavirus has been considered to be associated with a specific reservoir host, there have been cases that suggest rodent host expansion (e.g., pathogens that can infect multiple host species) for both HTNV and SEOV in China [22,118–120]. This challenges the strict rodent–virus coevolution theory and demonstrates that at least some hantaviruses can infect other susceptible rodents in addition to primary hosts, expanding the number of potential animal hosts [121]. These cases raise the question as to what role undetected or potential hosts play in hantavirus ecology and further influence the risk of human infections—a question largely ignored in the past and an area of further research. Further research is also needed to clarify the relationships between diversity and prevalence across types of ecosystems and host species, in particular, as the dilution effect on prevalence dynamics has broad potential applicability in predicting virus prevalence among rodent hosts.

To the best of our knowledge, this is the first systematic review to compare hantavirus disease dynamics, from environmental variability to rodent reservoir and to public health, among the main endemic areas across the globe. This review is timely because of the increasing public awareness of hantavirus diseases in Europe, in particular, over the last years. Limitations of this systematic review were that individual studies had differences—surveillance strategies for hantavirus infections, rodent sampling methods, rodent community compositions, socioeconomic factors, and environmental conditions. Therefore, studies were not all directly comparable, especially among China, European countries, and the US. Additionally, it should be noted that unusual human exposure (like war-induced exposure) to otherwise “normal” environment is an often forgotten risk factor for limited outbreaks [122]. Besides, there are more recent studies on ANDV-like viruses in South America that explore the environment conditions relevant to the occurrence of the host and the circulation of the virus, scrutinize the evidence for climate sensitivity of related disease risk, and recognize those areas of high risk for humans [91,123–126]. However, in this review, more attention was paid to longitudinal studies of sufficient scale. Finally, our focus on China in the review reflects the fact that for this country highly comprehensive data exist.

Establishing a mechanistically determined predictive framework for rodent-borne hantavirus disease prediction and prevention is urgently required to proactively protect the public from the increasing threat of hantaviruses. Such a framework would also provide insight into climate change, landscape alteration, rodent community composition, and pathogen spillover.

**Key Learning Points**

With this review we have demonstrated the following:

1. The complex seasonality and interannual cycles of hantavirus disease dynamics are a direct result of the (nonlinear) interaction between the population dynamics of the rodent host, environmental forcing, and human–wildlife contact patterns.
2. Specific environmental conditions can trigger hantavirus disease outbreaks, but the outcomes may differ among strains or areas mediated by the underlying mechanisms of hantavirus transmission. For example, in Weihe Plain of Central China, higher summer temperatures were followed by a lower incidence of HFRS in autumn; conversely, in North America, a positive relationship between hantavirus disease incidence and temperature was reported, indicating the complexity across different systems.
3. Current efforts to curb hantavirus transmission focus on avoiding contact between humans and host rodents as there is no WHO-licensed vaccine against hantavirus infections available (except Hantavax, which is licensed for human use only in the Republic of Korea). Hence, a broader appreciation for the epidemiological links among human beings, animals, and environment can result in more effective control of disease outbreak.

**Top Five Papers**

1. Yates TL, Mills JN, Parmenter CA, Ksiazek TG, Parmenter RR, et al. (2002) The ecology and evolutionary history of an emergent disease: hantavirus pulmonary syndrome. Bioscience 52: 989–998.
2. Glass GE, Yates TL, Fine JB, Shields TM, Kendall JB, et al. (2002) Satellite imagery characterizes local animal reservoir populations of Sin Nombre virus in the southwestern United States. Proc Natl Acad Sci USA 99: 16817–16822.
3. Clement J, Vercauteren J, Verstraeten W, Ducoffre G, Barrios J, et al. (2009) Relating increasing hantavirus incidences to the changing climate: the mast connection. International Journal of Health Geographics 8: 1.
4. Jonsson CB, Figueiredo LTM, Vapalahti O (2010) A global perspective on hantavirus ecology, epidemiology, and disease. Clinical Microbiology Reviews 23: 412–441.
5. Yan L, Fang LQ, Huang HG, Zhang LQ, Feng D, et al. (2007) Landscape elements and Hantaan virus-related hemorrhagic fever with renal syndrome, People's Republic of China. Emerging Infectious Diseases 13(9): 1301–1306.
